# Metastatic Melanoma to the Parotid Gland: A Case Report and Review of Current Diagnostic and Treatment Approaches

**DOI:** 10.7759/cureus.88892

**Published:** 2025-07-28

**Authors:** Dimitra G Simou, Lentiona Basiari, Evangeli Lampri, Alexandros Georgolios, Meropi Katsipaneli, Georgios V Psychogios

**Affiliations:** 1 Department of Otorhinolaryngology, Head and Neck Surgery, University Hospital of Ioannina, Ioannina, GRC; 2 Department of Pathology, University Hospital of Ioannina, Ioannina, GRC

**Keywords:** fine-needle aspiration, head and neck surgery, malignant melanoma metastasis, neck dissection, parotid melanoma, spindle cell melanoma, ultrasound elastography

## Abstract

Metastatic melanoma to the parotid gland is rare and represents a significant diagnostic challenge due to its atypical presentation, often resembling benign conditions, resulting in delays in diagnosis. Early and accurate detection is crucial for optimizing patient outcomes. We report the case of a 27-year-old woman who presented with a slowly growing, painless mass in her right parotid gland, which had been enlarging over the past three months. Her past medical history included the excision of an atypical blue nevus located on the scalp six years earlier. Imaging studies, including ultrasound and MRI, revealed an oval lesion in the right parotid gland's superficial lobe and ipsilateral intraparotid lymph nodes. Fine-needle aspiration cytology (FNAC) raised high suspicion of spindle cell melanoma, leading to an urgent total parotidectomy with selective neck dissection. Histopathological examination confirmed the diagnosis of metastatic melanoma. This case underscores the importance of considering melanoma metastasis in the differential diagnosis of parotid gland lesions, particularly in patients with a history of atypical pigmented skin lesions. A multimodal diagnostic approach, integrating FNAC, imaging, and histopathology, is essential for appropriate surgical planning. Long-term monitoring is essential to detect recurrence and metastasis, as melanoma is associated with locally aggressive behavior and a tendency for distant spread.

## Introduction

Metastatic melanoma to the parotid gland is a rare condition, accounting for a small percentage of salivary gland tumors [1]. Among all salivary gland tumors, around 80% arise in the parotid gland, and of these, approximately 25% are malignant [2]. While primary salivary gland melanoma is exceedingly rare, metastatic disease is more common, typically arising from cutaneous lesions of the scalp, face, or neck [3]. Metastatic melanoma to the parotid gland is particularly challenging to diagnose due to its often atypical presentation. It can sometimes resemble benign conditions, leading to delays in diagnosis. This highlights the importance of considering melanoma in the differential diagnosis of any parotid mass, particularly in patients with a history of pigmented skin lesions [1].

We present a rare case of parotid gland metastatic melanoma arising from a previously excised atypical blue nevus. The case emphasizes the diagnostic role of clinical history, imaging, and cytology, as well as the importance of multidisciplinary management in ensuring timely and effective treatment.

## Case presentation

A 27-year-old woman presented to our department in October 2024, complaining of a slowly growing, painless mass in her right parotid gland, which she had noticed over the past three months. She was a non-smoker, and the rest of her past medical history was unremarkable.

On physical examination, a soft, mobile, and well-circumscribed mass was palpated in the right preauricular region, without overlying skin changes or signs of inflammation. The lesion was not fixed to adjacent structures, and there was no facial nerve weakness or asymmetry. The remainder of the clinical examination was normal.

**Figure 1:**
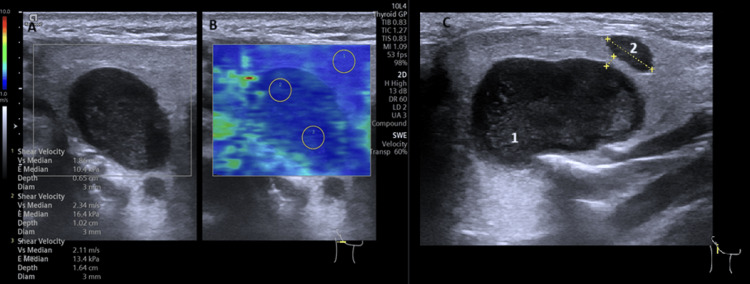
(A) B-mode ultrasound image showing a hypoechoic lesion with regular borders in the superficial lobe of the right parotid gland. (B) VTIQ of the same tumor indicating low stiffness. (C) Intraparotid tumor (1) and a secondary adjacent smaller hypoechoic nodule (2). VTIQ: Virtual Touch Imaging Quantification Elastography.

An ultrasound (U/S) examination using a linear 10 MHz probe revealed two hypoechoic lesions with regular borders in the superficial lobe of the right parotid gland: a larger one (16 x 12 x 23 mm) located centrally and closely related to the retromandibular vein, and a smaller, oval nodule located 2 mm inferiorly. Virtual Touch Imaging Quantification Elastography (VTIQ) indicated that both lesions were soft and homogeneous, suggesting a benign nature resembling pleomorphic adenoma (Figure 1) [4].

MRI further revealed an oval lesion in the right parotid gland with high signal intensity on T2-weighted images and intermediate signal on T1-weighted images, suspicious for pleomorphic adenoma, along with enlarged intraparotid lymph nodes. During a second outpatient visit, U/S-guided fine-needle aspiration cytology (FNAC) was performed, as per our department's routine preoperative protocol. Two weeks after the initial evaluation, FNAC confirmed a diagnosis of spindle cell melanoma (Milan VI). The patient was scheduled for surgery within two days of receiving the results. Upon specific questioning, she disclosed that in 2018 she had undergone a minor surgical procedure under local anesthesia for excision of a scalp lesion. Histopathological examination at that time revealed an atypical blue nevus of uncertain malignant potential, with free peripheral but close deep margins. No re-excision was performed. She was subsequently referred to a dermatologist and ophthalmologist, both of whom reported no pathological findings.

**Figure 2:**
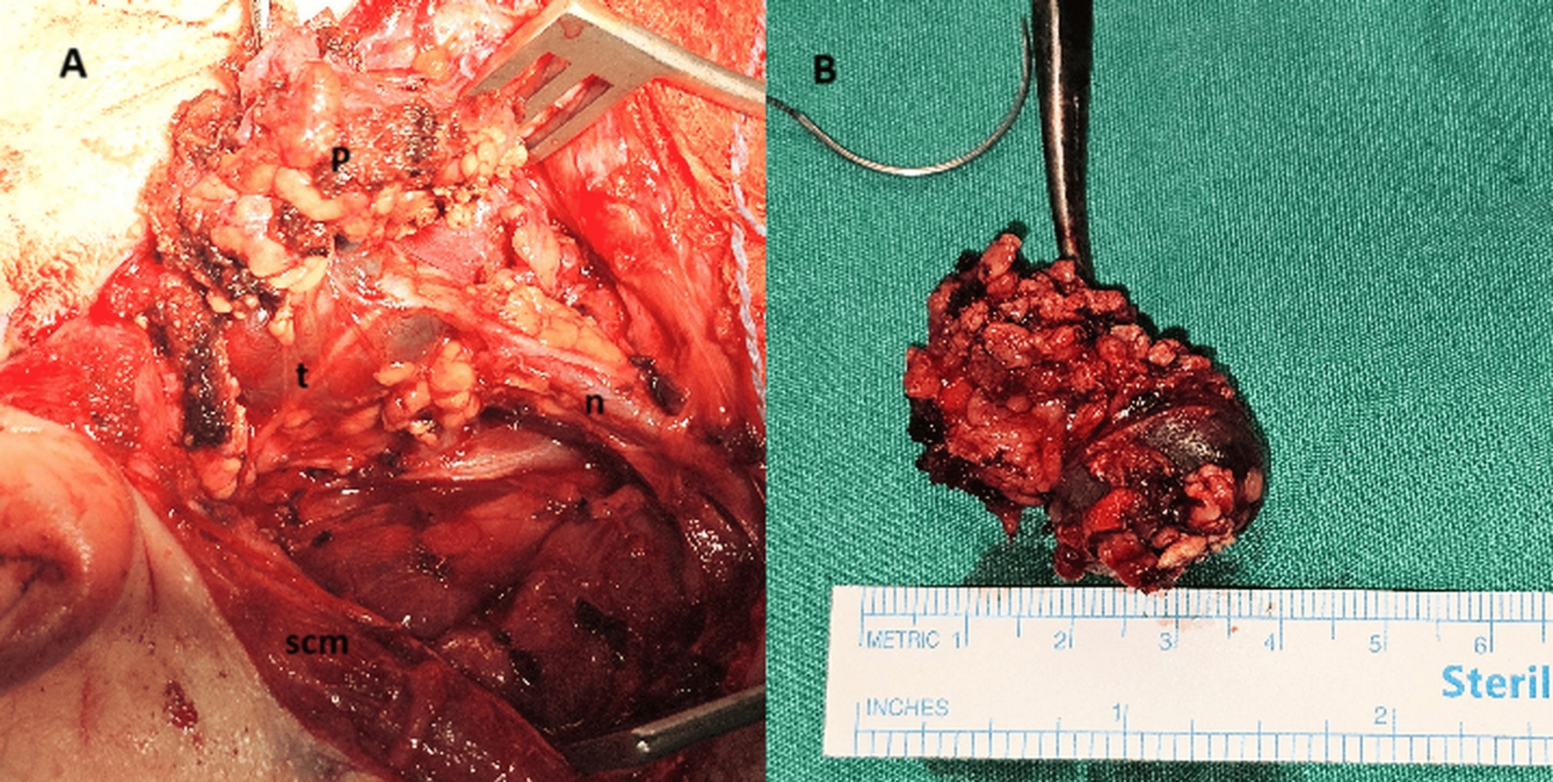
(A) Intraoperative view showing dissection of the parotid gland. Superficial parotid lobe (p), tumor (t), facial nerve (n), and sternocleidomastoid muscle (scm) are indicated. (B) Excised parotid tumor.

Following admission, a preoperative U/S revealed a third nodule not seen on the previous scan, as well as a non-suspicious cervical lymph node in right level IIb. Under general anesthesia and intraoperative neuromonitoring, a total parotidectomy with selective neck dissection (levels II and III) was performed. Intraoperatively, macroscopically pigmented lesions were identified within the parotid gland (Figure 2).

A meticulous dissection of the facial nerve branches was performed, and one small branch of the facial nerve and the retromandibular vein were resected because of their proximity to the larger tumor. Postoperatively, the patient had a House-Brackmann grade I function of the facial nerve.

**Figure 3:**
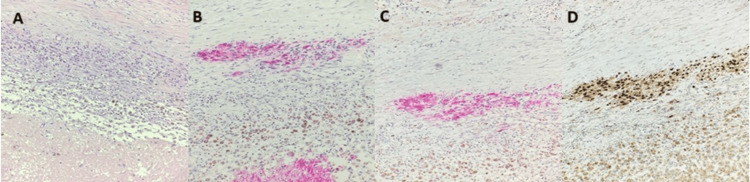
Histopathological and immunohistochemical sections from the excised parotid gland lesion. (A) H&E stain (20×) showing spindle-shaped malignant cells. (B) Immunohistochemical staining positive for HMB-45 (20×). (C) Positive immunostaining for MART-1/Melan-A (MIB) (20×). (D) Nuclear immunoreactivity for SOX10 (20×).

Histopathological examination confirmed spindle cell melanoma. Microscopic analysis revealed a malignant neoplasm with spindle-shaped cells, nuclear pleomorphism, and prominent nucleoli. Immunohistochemical staining showed strong and diffuse positivity for melanocytic markers (HMB-45, S100, SOX10) (Figure 3).

**Figure 4:**
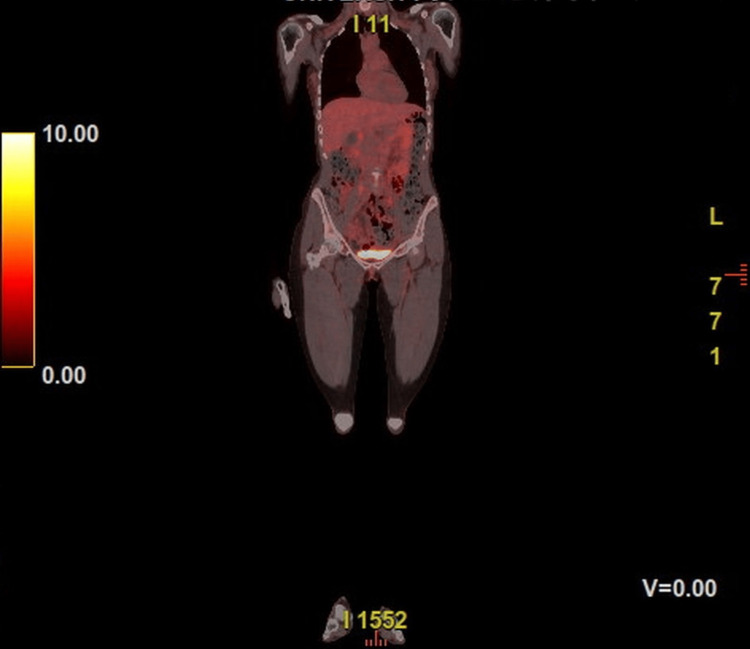
Coronal PET/CT scan performed postoperatively showing no evidence of distant metastatic disease.

Molecular analysis revealed the absence of the BRAF V600E mutation. R0 resection was difficult for the pathologist to confirm due to the presence of multiple melanoma foci within the parotid gland, which could not be resected en bloc, as this would have necessitated excision of the facial nerve. A postoperative PET/CT scan was scheduled for staging, and no evidence of distant metastases was found (Figure 4).

Therefore, based on the eighth edition of the American Joint Committee on Cancer (AJCC) melanoma staging system, the disease was classified as stage III [5].

The patient was referred to an oncological center for melanoma treatment and initiated adjuvant immunotherapy with nivolumab, given the absence of a BRAF mutation. Radiotherapy was not indicated due to the lack of extranodal extension or positive margins. An additional follow-up PET/CT scan was scheduled four months postoperatively, which also revealed no signs of recurrence or distant metastases.

## Discussion

Parotid gland melanoma is a rare occurrence, typically representing metastatic spread from a primary facial, scalp, or neck cutaneous lesion [2,3]. The patient's history of an atypical blue nevus, despite previous excision, highlights the risk of malignant transformation and the importance of meticulous follow-up for such lesions [1]. Adequate oncologic margins are crucial during any surgical resection of suspected melanoma.

Interestingly, some patients present with parotid melanoma without a known history of cutaneous lesions; in such cases, reviewing old photographs may reveal a previously existing, spontaneously resolved head or neck lesion as a potential primary source [2,6]. In situations where the primary lesion remains unknown, thorough dermatological, ophthalmological, and otolaryngological examinations are crucial to identify the primary site, as well as proctoscopy or sigmoidoscopy and gynecological examination [1].

As a first diagnostic step, U/S has become a standard imaging modality for evaluating salivary gland neoplasms, helping to identify malignant features such as heterogeneous echostructure and poorly defined margins. Shear wave elastography further enhances the assessment of tumor stiffness by measuring shear wave velocity (SWV) [4,7,8]. Malignant parotid tumors usually exhibit significantly higher SWV values (typically >6.0 m/s) compared to benign lesions (typically <3.5 m/s), demonstrating high sensitivity and specificity for differentiating between benign and malignant lesions [4,8]. In our patient, U/S revealed two hypoechoic lesions; elastography indicated a soft consistency, a finding inconsistent with the increased stiffness usually observed in malignant parotid tumors [7]. This contradictory finding emphasizes the risk of false-negative results when using elastography alone for definitive diagnosis, highlighting the need for a multi-modal approach. MRI provides complementary information, particularly in cases where U/S findings are inconclusive. However, in our case, MRI results were consistent with USG, revealing an oval lesion in the right parotid gland with high signal intensity on T2-weighted images and intermediate signal on T1-weighted images, findings suggestive of a pleomorphic adenoma rather than metastatic melanoma [9]. Melanotic lesions usually demonstrate high signal intensity on T1-weighted images due to the paramagnetic effect of melanin and a relatively reduced signal on T2-weighted images [9]. Preoperative U/S and MRI have demonstrated similar sensitivity, specificity, and accuracy in distinguishing benign from malignant parotid tumors [10]. However, U/S is operator-dependent and has limitations, particularly in assessing tumors located in the deep lobe due to the acoustic shadow of the mandibular ramus. This can lead to diagnostic challenges, especially in small or low-grade malignant tumors that may mimic benign features. Additionally, inflammatory conditions can further complicate interpretation.

FNA, especially when performed under US guidance, is very helpful in guiding decision-making. Moreover, in this case, FNA was of vital importance, as it led to the immediate diagnosis and the decision for surgical intervention. FNAC is a highly accurate diagnostic tool for evaluating parotid gland lesions. A systematic review and meta-analysis demonstrated that FNAC has a sensitivity of 96% and specificity of 98% in differentiating neoplastic from non-neoplastic lesions, and an 80% sensitivity and 97% specificity in distinguishing malignant from benign tumors [11]. Similarly, FNAC has been reported to have a sensitivity and specificity of 75% and 95% for parotid malignancies, highlighting its reliability despite a non-diagnostic rate of 8.6%-17.8% [12].

Surgical management, given the multifocality and potential for cervical lymph node involvement, consisted of a total parotidectomy with selective neck dissection (levels II and III) [6,13]. The decision to perform a total parotidectomy with neck dissection is supported by the observation that patients with cutaneous malignancies metastatic to the parotid superficial lymph nodes frequently have occult metastasis in the deep lobe and/or cervical lymph nodes [14]. In addition, parotid deep lobe metastasis and cervical metastasis often occur in the same patients, with both representing a more advanced and aggressive cancer. Therefore, total parotidectomy with ipsilateral neck dissection is recommended to optimize regional control [14].

Further supporting this approach, a retrospective cohort study found that metastatic disease is frequently present in lymph nodes deep to the facial nerve when total parotidectomy is performed for metastatic melanoma or other parotid malignancies. Moreover, literature reports a 7% recurrence rate in the parotid bed following superficial parotidectomy, whereas total parotidectomy is associated with a 0% recurrence rate, reinforcing its role in achieving better regional disease control. Local recurrence has been associated with a high rate of facial nerve involvement and poor quality of life due to severe pain [15].

Treatment decisions regarding the extent of neck dissection depend on the primary lesion's location, potentially involving selective neck dissection levels I-IV for preauricular lesions, levels II-V for postauricular lesions, or levels I-V for lesions in the scalp's watershed region between the ears [6,16]. Suspicion of metastatic involvement of the parotid tail may necessitate the inclusion of level V dissection [16].

Facial nerve sacrifice in parotidectomy for metastatic melanoma is not well documented, but data from cutaneous squamous cell carcinoma metastatic to the parotid suggest that 27-40% of cases require nerve resection, primarily due to extracapsular extension. When margins are close, wide excision reduces recurrence risk, though nerve preservation may be considered with adjuvant radiotherapy. Additionally, metastases in the gland parenchyma and signs of perineural invasion increase the likelihood of nerve involvement, aiding in surgical planning and preoperative patient counseling [17].

Adjuvant treatment for metastatic melanoma depends on disease extent, lymph node involvement, and molecular characteristics. Given the absence of a BRAF mutation, our patient received Nivolumab, a PD-1 inhibitor, as the preferred immunotherapy [6,18,19].

PD-1 inhibitors such as Nivolumab and Pembrolizumab enhance the immune response against tumor cells by blocking the interaction of PD-1 with its ligands. Clinical trials have demonstrated that Pembrolizumab significantly improves recurrence-free survival (RFS) in high-risk stage III melanoma, making it a key adjuvant option [18,20].

Ipilimumab, a CTLA-4 inhibitor, can be combined with Nivolumab to further enhance anti-tumor immunity for patients with advanced disease. However, while this combination achieves higher pathologic response rates, it is associated with significant toxicity, making Nivolumab monotherapy the standard choice in cases where toxicity is a concern [18].

For BRAF V600-mutant melanoma, targeted therapy with Dabrafenib/Trametinib offers survival benefits, as BRAF-mutant patients exhibit higher recurrence rates and worse disease-specific survival than wild-type cases [19,21]. However, in BRAF wild-type cases like ours, immunotherapy remains the first-line option [18,19,21].

Talimogene laherparepvec (T-VEC), an oncolytic virus therapy, is primarily used for in-transit metastases rather than nodal or distant metastatic disease, and was therefore not considered in this case [18].

Ultimately, the choice of adjuvant therapy is individualized, balancing efficacy and toxicity. In this case, Nivolumab monotherapy was selected for its proven benefit in resected, BRAF wild-type stage III melanoma, offering effective disease control with manageable side effects [18,20,21].

Adjuvant radiotherapy is not routinely recommended for regionally metastatic head and neck melanoma, as overall survival is primarily dictated by distant metastases. However, studies suggest it may be beneficial in patients with extracapsular spread, improving locoregional control by reducing cervical recurrence from 14% to 7%. While melanoma has traditionally been considered radioresistant, there is evidence indicating that with appropriate fractionation (e.g., 6 Gy × 5 fractions), locoregional control rates of up to 90% can be achieved, particularly after limited neck dissection. Despite these benefits, radiotherapy has not been shown to improve overall survival in these patients [22-24].

Long-term follow-up is essential to monitor for recurrence and detect distant metastases, given the potential for melanoma to metastasize to distant sites like the bladder, bowel, and brain [25]. According to current guidelines, patients with stage III melanoma should undergo physical examinations every 3 to 6 months during the first two years, then every 3 to 12 months for the next three years, followed by annual evaluations as clinically indicated [26]. Imaging studies such as PET/CT or MRI may also be used during the first five years for early detection of recurrence, especially in high-risk patients [26]. Multiple factors, including the stage of the disease, lymph node involvement, and response to therapy, determine the prognosis [5,13,25].

## Conclusions

Metastatic melanoma of the parotid gland is a rare but clinically significant condition that presents unique diagnostic and therapeutic challenges. This case underscores the importance of thorough evaluation, including imaging studies and fine-needle aspiration cytology, for the accurate diagnosis and effective management of parotid masses. The patient's history of an atypical blue nevus highlights the need for careful long-term monitoring of previously excised pigmented lesions, as they may carry a risk of malignant transformation.

Surgical management, particularly total parotidectomy with selective neck dissection, is recommended to achieve adequate oncologic control and address potential lymph node involvement. In addition to surgery, adjuvant systemic therapy should be considered based on disease stage, nodal involvement, and molecular characteristics, as it plays a critical role in improving long-term outcomes. A multidisciplinary approach, engaging clinicians, oncologists, radiologists, and pathologists, is essential for devising an effective treatment strategy and ensuring optimal patient care. Long-term follow-up remains crucial for early detection of recurrence or distant metastases, given melanoma’s aggressive nature and potential to spread to distant organs. Future research should aim to refine diagnostic pathways and expand therapeutic options to enhance outcomes for patients affected by this challenging malignancy.
